# Phylogenomic Analysis of Human Papillomavirus Type 31 and Cervical Carcinogenesis: A Study of 2093 Viral Genomes

**DOI:** 10.3390/v13101948

**Published:** 2021-09-28

**Authors:** Maisa Pinheiro, Ariana Harari, Mark Schiffman, Gary M. Clifford, Zigui Chen, Meredith Yeager, Michael Cullen, Joseph F. Boland, Tina Raine-Bennett, Mia Steinberg, Sara Bass, Yanzi Xiao, Vanessa Tenet, Kai Yu, Bin Zhu, Laurie Burdett, Sevilay Turan, Thomas Lorey, Philip E. Castle, Nicolas Wentzensen, Robert D. Burk, Lisa Mirabello

**Affiliations:** 1 Division of Cancer Epidemiology and Genetics, National Cancer Institute, National Institutes of Health, Rockville, MD 20850, USA; maisa.pinheiro@nih.gov (M.P.); mark.w.schiffman@gmail.com (M.S.); yeagerm@mail.nih.gov (M.Y.); michael.cullen@nih.gov (M.C.); bolandj2@mail.nih.gov (J.F.B.); mia.steinberg@nih.gov (M.S.); sara.bass2@nih.gov (S.B.); yanzi.xiao@nih.gov (Y.X.); yuka@mail.nih.gov (K.Y.); bin.zhu@nih.gov (B.Z.); burdettl@mail.nih.gov (L.B.); sevilay.turan@nih.gov (S.T.); philip.castle@nih.gov (P.E.C.); wentzenn@mail.nih.gov (N.W.); 2 Departments of Pediatrics and Microbiology & Immunology, Albert Einstein College of Medicine, Bronx, NY 10461, USA; abharari@gmail.com; 3 Early Detection, Prevention and Infections Branch, International Agency for Research on Cancer (IARC/WHO), 150 cours Albert Thomas, CEDEX 08, 69372 Lyon, France; cliffordg@iarc.fr (G.M.C.); tenetv@iarc.fr (V.T.); 4 Department of Microbiology, The Chinese University of Hong Kong, Hong Kong, China; zigui.chen@cuhk.edu.hk; 5 Cancer Genomics Research Laboratory, Leidos Biomedical Research, Inc., Frederick, MD 21701, USA; 6 Division of Research, Kaiser Permanente Northern California, Oakland, CA 94612, USA; tina.r.raine-bennett@kp.org; 7 Regional Laboratory, Kaiser Permanente Northern California, Oakland, CA 94710, USA; Thomas.Lorey@kp.org; 8 Division of Cancer Prevention, National Cancer Institute, National Institutes of Health, Rockville, MD 20850, USA; 9 Department of Epidemiology & Population Health, and Obstetrics, Gynecology and Women’s Health, Albert Einstein College of Medicine, Bronx, NY 10461, USA

**Keywords:** HPV31, cervical carcinogenesis, viral genomic variation, viral methylation

## Abstract

Human papillomavirus (HPV) type 31 (HPV31) is closely related to the most carcinogenic type, HPV16, but only accounts for 4% of cervical cancer cases worldwide. Viral genetic and epigenetic variations have been associated with carcinogenesis for other high-risk HPV types, but little is known about HPV31. We sequenced 2093 HPV31 viral whole genomes from two large studies, one from the U.S. and one international. In addition, we investigated CpG methylation in a subset of 175 samples. We evaluated the association of HPV31 lineages/sublineages, single nucleotide polymorphisms (SNPs) and viral methylation with cervical carcinogenesis. HPV31 A/B clade was >1.8-fold more associated with cervical intraepithelial neoplasia grade 3 and cancer (CIN3+) compared to the most common C lineage. Lineage/sublineage distribution varied by race/ethnicity and geographic region. A viral genome-wide association analysis identified SNPs within the A/B clade associated with CIN3+, including H23Y (C626T) (odds ratio = 1.60, confidence intervals = 1.17–2.19) located in the pRb CR2 binding-site within the E7 oncogene. Viral CpG methylation was higher in lineage B, compared to the other lineages, and was most elevated in CIN3+. In conclusion, these data support the increased oncogenicity of the A/B lineages and suggest variation of E7 as a contributing risk factor.

## 1. Introduction

Invasive cervical cancer (ICC), caused by human papillomavirus (HPV), is the 4th most common cancer diagnosed among women worldwide and the leading cause of cancer deaths in 42 countries [1]. Rates of ICC have been decreasing over the past decades mostly due to screening in more developed countries [2], with HPV vaccination expected to have a long-term impact on further reducing the burden of ICC [3]. However, there were still 570,000 new ICC cases and 311,000 deaths estimated worldwide in 2018 [1].

Over 200 HPV (geno)types have been characterized [4,5] (https://pave.niaid.nih.gov/, accessed on 8 January 2021), but only 13 high-risk (HR) types, from related phylogenetic clusters, are responsible for virtually all ICC [6,7,8]. HPV16 accounts for 60% of cases worldwide, while HPV31, sharing a most recent common ancestor (MRCA) and with about 70% DNA similarity to HPV16 [9], accounts for only 4% of cases [6,10]. This is particularly important because viral carcinogenicity reflects viral evolution but the exact genetic basis of this seemingly solvable problem is not known [7]. HPV31 prevalence varies by geographic region and is more common among ICC in North, Central and South America than other regions of the world [11,12].

Controlled by a cellular immune response, the majority of infections with HR-HPVs clear within two years [13,14,15], and persistent infections only sometimes progress and cause cancer [16]. HPV genotyping and HPV methylation have been shown to be promising strategies to detect infections that are more likely to progress to cervical precancer and cancer (cervical intraepithelial neoplasia grades 2 and 3, and cancer (CIN2/3+)) [17,18]. Previous studies have shown that cervical carcinogenesis is linked to genomic variation within an HPV type (e.g., HPV lineages, sublineages or single nucleotide polymorphisms (SNPs)); important differences in cervical precancer and cancer risk have been linked to viral lineages and sublineages [19,20,21,22,23], and to even finer levels of viral variation at the nucleotide level [24]. For HPV16, the D2 sublineage has been associated with a 28-fold increase in ICC (95% CI = 9.27 to 87.55, *p* = 5.0 × 10^−9^) compared to the more common sublineages, A1/A2 [21], and hypovariation of the E7 oncogene is linked to HPV16 carcinogenesis [24]. Less is known about the genomic variation of HPV31. HPV31 has three main lineages, A, B and C [20,25], and two small studies reported that the A and B lineages were associated with precancer/cancer compared with HPV31 C lineage [19,23].

Viral DNA methylation (at CpG sites) has been positively associated with cervical precancer/cancer across HR-HPV types, and high levels of methylation at specific CpG sites have been shown to predict infections progressing to CIN2/3+ [18,26,27,28,29]. For HPV31, studies using pyrosequencing [27,28,30,31,32] and next generation sequencing [18] assays have demonstrated an association of higher methylation levels at specific CpG sites with precancer/cancer compared with infections that did not progress. Despite HPV lineages and methylation levels both being associated with differences in oncogenic risk, the relationship between HPV lineages and methylation has not been adequately evaluated for the HR-HPV types.

The goal of this investigation was to interrogate the HPV31 genome to discover features of the genetic and epigenetic variations associated with cervical carcinogenesis by whole genome sequencing 2093 HPV31-positive cervical samples from the U.S. NCI-Kaiser persistence and progression (PaP) study and from the multi-country international collection from the International Agency for Research on Cancer (IARC) collection. This is the largest study of HPV31 genomes to date, and we additionally assess viral methylation across evolutionary derived HPV31 lineages.

## 2. Materials and Methods

### 2.1. Study Population

#### 2.1.1. PaP Study

We designed an HPV31 nested case-control study within the PaP cohort at Kaiser Permanente Northern California (KPNC), in the U.S. The PaP study has been previously described [33], and included approximately 55,000 women, aged 21 to 70 years, that underwent routine cervical cancer screening between December 2007 to January 2011, using cytology (first specimen) and HPV (second specimen) cotesting. This study was designed to evaluate HPV genotypes and other risk markers in cervical exfoliated cells across the cervical multi-step carcinogenesis model, including HPV infection, precancer and cancer, in a large number of women in the U.S. [34,35]. Residual cervical specimens from liquid-based cytology and de-identified clinical information such as age at diagnosis, self-reported race/ethnicity, follow-up cytology and pathology were obtained from electronic medical records. Cervical exfoliated cells were tested clinically using Hybrid Capture 2 (HC2; Qiagen Inc., Gaithersburg, MD, USA), which is capable of detecting 13 HR HPV types. Typing of archived specimens in neutralized specimen transport medium (STM; Qiagen Inc., Gaithersburg, MD, USA) was performed using a variety of assays, including Onclarity (BD, Franklin Lakes, NJ, USA), Linear Array (Roche Diagnostics, Indianapolis, IN, USA) or MY09-MY11 PCR based on prior sub-studies.

Outcome ascertainment was completed in 2017. Cervical specimens were categorized according to corresponding histology results, as cervical intraepithelial neoplasia (CIN) grade 1 (CIN1), CIN grade 2 (CIN2), CIN grade 3 (CIN3), adenocarcinoma in situ (AIS) or invasive cervical cancer (ICC) including squamous cell carcinoma, adenocarcinoma and adenosquamous carcinoma. In this nested-case control study, “cases” were defined as women positive for HPV31 diagnosed with CIN2+ (CIN2, CIN3/AIS or ICC), and “controls” were defined as women positive for HPV31 diagnosed with CIN1 or lower (within normal limits (WNL) or atypia), who subsequently cleared their HPV31 infection or did not progress to CIN2+ throughout the study follow-up (from 2007 to 2017). Samples were collected prior to or at the time of CIN2+ diagnosis and the mean time between the tested samples and CIN2+ diagnosis was 1.07 years. If women self-reported their ethnicity as Hispanic, they were classified as Hispanic. Women not classified as Hispanic were assigned according to their reported race: White, Black (includes African-American, and referred to in this manuscript as African-American), Asian (includes Hawaiian/Pacific Islander) or other (including multiple races selected).

In total, 2073 cervical specimens testing positive for HPV31 were evaluated, including all available 787 CIN2+ cases (7 ICC, 333 CIN3, 9 AIS, 438 CIN2) and 1286 controls randomly selected (Figure S1). HPV31 infections were either single or coinfected with other oncogenic types. The National Cancer Institute and Kaiser Permanente Institutional Review Boards approved this study. Women were mailed information on the study and could opt-out of inclusion.

#### 2.1.2. IARC Study

Our second sample set was obtained from the IARC as part of their coordinated studies conducted to understand the worldwide HPV genotype distribution, using cervical cytology samples and frozen or formalin fixed paraffin-embedded (FFPE) tissues samples [36,37,38,39]. Samples were collected by IARC from women with and without cervical cancer in 32 countries around the world. Cervical samples were genotyped using general primer GP5+/6+-mediated PCR with enzyme immunoassay and a subsequent genotyping readout was used to detect and genotype HPV DNA [40].

In total, we included 628 HPV31-positive samples from IARC, including 147 cervical cancers and 481 non-cervical cancers (Figure S1). This study was approved by IARC ethical committees (IARC ICE 07/40 approved on 21 December 2007).

### 2.2. DNA Isolation, Library Construction and Next-Generation Sequencing

For the PaP study samples, DNA was extracted using proteinase K, where 30 µL of the banked STM cells were transferred to 100 µL of K buffer containing 200 µg/mL proteinase K and incubated at 55 °C for 2 h followed by a 10-min incubation at 95 °C [41]. For the IARC samples, samples containing cervical cells and/or tissue were collected, and DNA was extracted according to previous study protocols [36,37,38,39,42]. Then, DNA underwent library construction protocol according to the manufacturer’s recommendation, using AmpliSeq Library Preparation kit 2.0-96LV (Thermo Fisher Scientifics, Waltham, MA, USA, Part #4480441) and custom oligonucleotide primers, designed by Life Tech in conjunction with our lab personnel, that amplify 44 overlapping amplicons covering 100% of the HPV31 viral genome. Library preparation was performed following the manufacturer’s protocol with detailed modifications described previously [43]. Briefly, DNA underwent two separate amplification reactions for 2 sets of non-overlapping primers targeting only the HPV31 whole-genome, for a total of two amplifications per sample. The two PCR reactions were then combined for sequencing barcode-adapters ligation. Amplification was performed using Phusion High-Fidelity DNA Polymerase (Thermo Fisher Scientifics, Waltham, MA, USA), with an error rate less than 1%. Individual libraries were quantified prior to sequencing using the Kapa Biosystems Library Quantification Kit-IonTorrent/LightCycler 480 (Roche, Basel, Switzerland), and library concentration was determined using Agilent BioAnalyzer DNA High-Sensitivity LabChip (Agilent Technologies, Santa Clara, CA, USA). Average amplicon size was 300 bp. Up to 96 samples were pooled on Ion 540 chips for high throughput sequencing on a Thermo Fisher Life Science Ion Torrent S5 GeneStudio system (Thermo Fisher Scientifics, Waltham, MA, USA) and a total of 60–80 million reads per chip was routinely obtained.

Raw sequence reads were quality assessed and trimmed, and then mapped to the 7912 bp HPV31 reference genome, GenBank accession no. J04353.1 (Table S1), using Ion Torrent Suite software (Thermo Fisher Scientifics, Waltham, MA, USA). One of the amplicons was split bioinformatically to enable mapping of the HPV circular DNA to the linear reference genome. An in-house developed pipeline was used for variant calling and gene annotation using the Torrent Variant Caller v.5.0.3 and snpEff v.3.6c [44]. Settings and the detailed pipeline are available at https://github.com/NCI-CGR/cgrHPV31, accessed on 9 September 2021. These analyses were executed using Snakemake [45].

### 2.3. Viral Methylation Assay

A total of 175 HPV31 single type infections were randomly selected, including 89 CIN3+ cases and 86 controls. Briefly, 1 μL of bisulfite converted DNA was amplified using Pyromark reagents (Qiagen, Valencia, CA, USA). HPV31 NGS methylation barcoded primers were designed [46] for use on the Illumina HiSeq 2000. Primers for three HPV31 methylation assays were developed targeting 22 CpG sites in the E2, L2 and L1 ORFs as reported [30] (Table S2). All primers were synthesized by IDT (Integrated DNA Technologies, Coralville, IA, USA). Barcoded PCR products were pooled at approximately equal concentrations and purified by DNA electro-elution and isopropanol precipitation or using the QIAquick Gel Extraction Kit (Qiagen). Library preparation was performed with purified PCR products using the KAPA LTP Library Kit (Kapa Biosystems, Wilmington, MA, USA) and paired-end 100 bp Illumina HiSeq2000 sequencing (Illumina Inc., San Diego, CA, USA) at the Albert Einstein College of Medicine, Genomics Core Facility. Samples were combined in pools for sequencing based on an estimated average depth of 2000 reads per sample.

A bioinformatic pipeline included demultiplexing of NGS reads using in-house scripts. Briefly, a bisulfite modified HPV31 reference sequence containing non-CpG cytosine as thymine was set as the reference sequence for global methylation alignment of all reads in bowtie v0.12.9 [47]. The percentage of reads containing either a “C” or “T” at a CpG site was calculated and a methylation percent was determined using Bismark v0.7.7 [48], with a quality score parameter set to ≥Q30. CpG site methylation proportion or percent (%) was identified based on the ratio of reads having “C” or “T” at the targeted CpG site.

Methylation PCR bias was controlled for by designating two control samples per HPV31 variant lineage (A, B, C), with prior known methylation results [30], which were used as positive controls, and water blanks processed through the whole procedure were included as negative controls. The positive controls allowed us to assess conversion efficiency by documenting expected percent methylation as previously reported [30].

### 2.4. Statistical Analyses

#### 2.4.1. HPV31 Lineage Assignment

Samples were considered for further phylogenetic analyses if they had a minimum HPV genome coverage of ≥1700 nucleotides and ≥4 mean reads per amplicon. Individual nucleotide positions were included in the analyses if covered by at least 4 sequence reads (i.e., ≥ 4x). After applying these coverage filtering criteria, a total of 297 (14.3%) and 109 (17.4%) samples were excluded from the PaP and IARC samples, respectively (Figure S1). Subsequently, a consensus sequence FASTA file was built for each individual sample and polymorphic sites were incorporated in the sequence if they were present in more than 60% of the reads. The average FASTA sequence length (i.e., nucleotide positions) covered with ≥ 4x was 7123.9 (median: 7484) (Table S3). Next, we combined our sequence FASTA files with lineage/sublineage reference sequences obtained from GenBank (Table S1) and built two phylogenetic trees with RAxML MPI [49] (options: raxmlHPC-MPI -f a -m GTRCAT) and MEGA7 [50] (Neighbor Joining, Model: p-distance and pairwise deletion), with 1000 bootstraps. Trees were visualized with MEGA7 [50] and ITOL [51]. Lineages/sublineages were assigned by visual inspection of the trees by proximity to the reference genomes. Lineage defining SNPs were visualized with the integrative genomics viewer [52]. Each sample was classified as one of the HPV31 evolutionary derived lineages—A, B or C—and sublineages—A1, A2, B1, B2, C1, C2, C3 or a new C4 sublineage.

#### 2.4.2. Statistical Analysis of Viral Genetic Variation

Differences in the HPV31 lineage distribution across histology, race/ethnicity and geographic regions were evaluated by Chi-Square and Fisher’s exact tests. Associations of HPV31 lineages and individual SNPs with CIN2+/CIN3+, compared to controls, were assessed using logistic regression to obtain odds ratios (OR) and 95% confidence intervals (CI). We also investigated potential confounders such as smoking, body mass index and age, and none were associated or impacted the strength of the outcome associations, therefore we are presenting the unadjusted models. For the lineage and sublineage analyses, we used the most common C lineage or C3 sublineage, respectively, as the referent group. For the race/ethnicity analyses, we excluded women who did not self-report race/ethnicity (*n* = 123) and women reporting multiple races/ethnicities (*n* = 15). We tested for effect modification of race/ethnicity on the associations between lineage and CIN2+ by stratification. For each lineage, we assessed the association of one race/ethnicity group with CIN2+, compared to women from all other races/ethnicities combined, as the referent group. The Wald-test was used to assess heterogeneity between ORs. For the individual SNP analyses, we used the most common nucleotide at each genomic position as the referent group, and significant *P* values were corrected for multiple comparisons using false discovery rate (FDR) based on the number of common polymorphic sites with minor allele frequency (MAF) > 1% (*n* = 199). To assess the burden of combined rare genetic variant sites by 10 genomic regions, we used fisher’s exact test and corrected *p*-values using FDR. To further investigate whether the burden of mutations was influenced by selective pressure in the PaP samples, we performed dN/dS analyses using SNPgenie [53], following the default parameters. For the PaP analysis, we excluded 199 samples with an HPV16 coinfection because of the predominant etiologic role of HPV16 among CIN2+ cases. Three samples from North America collected by the IARC were also excluded from the analysis because of the small sample size compared to other world regions. For the final analyses, we used 2093 samples from both the PaP Study and IARC collection (PaP *n* = 1577; IARC *n* = 516; Total *n* = 2093, Table S4 and Figure S1). A summary of HPV sequencing coverage and quality statistics of the next generation sequencing data is shown in Table S3. Statistical analyses were performed in R version 3.5.3. Case-control association analyses were not performed using IARC data due to uneven collection of cases and controls by region/country (Table S5, Figure S1). All statistical tests were two-sided.

#### 2.4.3. Statistical Analysis of Viral Methylation

In a subset of 175 HPV31-positive PaP samples, we assessed the methylation pattern of 22 CpG sites cross E2, L2 and L1 genes by lineages. CpG sites that were previously reported as highly different between CIN3+ cases and controls were selected for evaluation in our study [30]. We calculated median percent methylation and first compared the overall methylation levels between lineages by CIN3+ cases and controls; then, we compared values between cases and controls by site using Mann–Whitney U tests. For the case-control comparisons, methylation levels were categorized into tertiles, and the OR and 95% CI for CIN3+ vs. controls was calculated by comparing the highest tertile versus (vs.) the middle and low tertiles combined as the referent group. Receiver operating characteristic (ROC) curves and areas under the curve (AUC) with 95% CIs were calculated. *p* values were corrected for multiple comparisons using FDR. Statistical analyses were performed in R version 3.5.3. Here, all statistical tests were also two-sided.

## 3. Results

### 3.1. Distribution of HPV31 Lineages in PaP and IARC

**Figure 1:**
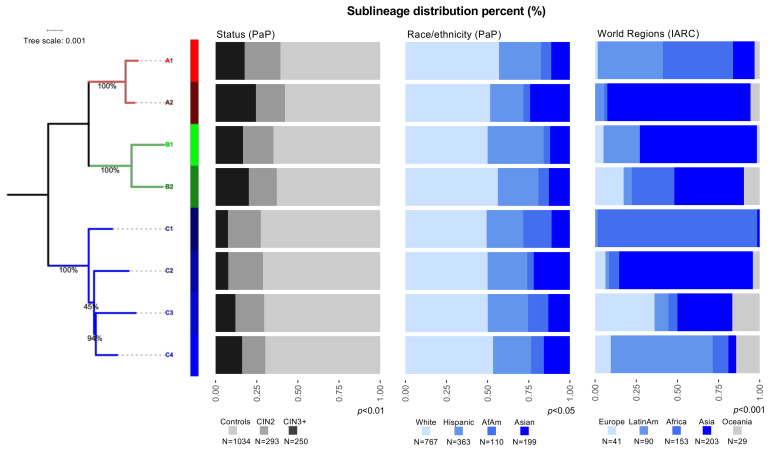
HPV31 phylogenetic tree of lineages and sublineages and the characteristics of each sublineage by case-control status and race/ethnicity are illustrated for the PaP Study and by world region for the IARC collection as indicated. Footnote. Controls = Cervical intraepithelial neoplasia (CIN) grade 1 or lower (≤CIN1); CIN2 = CIN grade 2; CIN3+ = CIN grade 3 and cancer; AfAm = African-American; LatinAm = Latin-American. The maximum likelihood (ML) tree was constructed using RAXML version 8.2.12, with bootstrap supports indicated on or near branches. Bar plots represent the percent of each corresponding sublineage by case-control status, race/ethnicity or world region as indicated with numbers of samples provided under the colored squares. *p* = Fisher’s exact test.

To investigate the association of viral genetic variation with cervical carcinogenesis, we assigned each HPV31 isolate to a specific lineage and/or sublineage using viral whole genome sequence information. In the PaP Study, using 1034 controls, 293 CIN2, 246 CIN3 and 4 ICC, HPV31 lineages significantly varied by case-control status (*p* < 0.01) and self-reported race/ethnicity (*p* < 0.05) (Figure 1; Table S6). The most common HPV31 lineage in this study was the C lineage (*n* = 738, 46.8%); whereas, for sublineages it was the C3 sublineage (*n* = 457, 29.0%), followed by A1 (*n* = 441, 28.0%) and B2 (*n* = 242, 15.3%) (Figure 1, Table S6). Compared to women from all other races/ethnicities, C1 was significantly more prevalent among African-American women (10.9% vs. 4.7%, *p* < 0.01), A2 and C2 were significantly more prevalent among Asian women (15.6% vs. 8.6%, *p* < 0.01), and A1 and B2 were significantly more prevalent among White women (45.3% vs. 39.4%, *p* = 0.03).

In 516 HPV31-positive samples collected by IARC, the distribution of lineages significantly varied by worldwide region (Figure 1; Table S6). Lineage C was more common in women from Africa (*n* = 70, 45.8%), predominantly driven by the C1 sublineage (*n* = 63, 41.2%) (Figure 1; Table S6). Moreover, dichotomizing groups by geography, sublineage C1 was significantly more prevalent in women from Africa (41.2% vs. 0.6%, *p* < 0.0001), A2 and C2 were significantly more prevalent in women from Asia (42.4% vs. 5.1%, *p* < 0.0001), A1 and B1 were significantly more prevalent in women from Latin-America (71.1% vs. 29.1%, *p* < 0.0001) and B2 was significantly more prevalent in women from Europe (43.9% vs.18.1%, *p* < 0.001).

Of note, we also identified a new sublineage, C4, that differed by 1.09% (±0.12%) from A lineages, 1.14% (±0.11%) from B lineages and 0.41% (±0.06%) from C1, 0.40% (±0.07%) from C2 and 0.32% (±0.05%) from C3 sublineages. The new C4 sublineage corresponded to 9.5% and 4.1% of samples from the PaP Study and IARC collection, respectively, and it was most commonly identified in women from Latin-America in the worldwide IARC collection (14.4%) (Figure 1; Table S6).

### 3.2. HPV31 Lineages Are Associated with Precancer and Cancer

Using the PaP nested case-control study, we assessed associations between each HPV31 lineage/sublineage and cervical precancer and cancer (CIN2+ and CIN3+ separately), compared to the most common lineage/sublineage, C/C3 (Table 1). The A (OR = 1.85, 95% CI = 1.35–2.54) and B (OR = 1.82, 95% CI = 1.25–2.63) lineages were associated with CIN3+. Taking it to a finer level of genetic variation, sublineages A1 (OR = 1.71, 95% CI = 1.17–2.50), A2 (OR = 2.48, 95% CI = 1.43–4.29) and B2 (OR = 1.89, 95% CI = 1.23–2.90) were significantly associated with CIN3+, compared to the C3 sublineage (Table 1). Sublineages A1, A2 and B2 were similarly associated with CIN3+ (OR range 1.90–3.47) among HPV31 single infections. Results were similar for CIN2+ (Table S7). The associations with CIN3+ for HPV31 lineages varied by a women’s race/ethnicity only for the A lineage (Table 1), and White women with HPV31 A had relatively significantly more CIN3+ (OR = 1.71, 95% CI = 1.07–2.72) compared to women from all other races/ethnicities. For CIN2+, associations were similar for White women with an A infection, however Hispanic women with an A or C infection had an inverse association with CIN2+ compared to all other races/ethnicities (Table S7). No specific sublineage was significantly associated with CIN3+ and a women’s race/ethnicity. For CIN2+, there was an increased association for White women infected with A1 or B2 sublineages, compared to women from other races/ethnicities (OR = 1.47, 1.06–2.04) (data not shown).

### 3.3. HPV31 Individual SNPs Are Associated with Cervical Carcinogenesis

**Figure 2:**
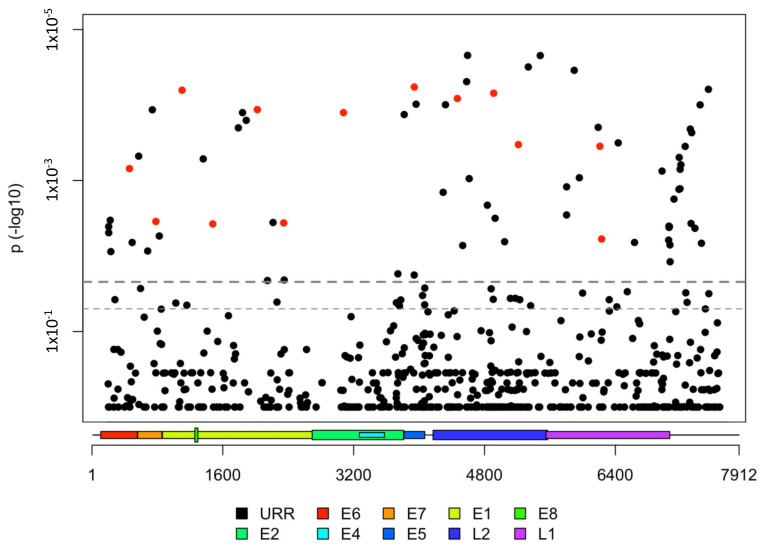
HPV31 viral genome wide association analysis and SNPs associated with CIN3+ in the PaP Cohort. Red circles indicate the 13 nonsynonymous SNPs significantly different between cases and controls after false discovery rate (FDR) correction for multiple comparisons. Dashed thicker line represents the FDR significance threshold. Dashed thinner line represents the logistic regression 0.05 significance threshold. *y*-axis represents *p*-values in logarithm scale. *x*-axis represents HPV31 genome positions and viral gene regions indicated by the colored key below the figure.

We further evaluated finer HPV31 genetic variation down to the nucleotide level (i.e., individual single nucleotide polymorphisms (SNPs) for associations with CIN3+ in the PaP Study. There was a total of 1143 polymorphic SNP sites amongst 1284 HPV31 genomes, 73.5% (*n* = 944) were rare (minor allele frequency (MAF) < 1%) and 15.5% (*n* = 199) were common (MAF ≥ 1%). For the common variation, 57 SNPs were significantly different between CIN3+ cases and controls after FDR correction for multiple tests (Figure 2, Table S8). Of these, 22.8% (*n* = 13) were nonsynonymous variants (Figure 2), and six were within motifs suggesting APOBEC3-induced mutations (Table S8). All SNPs associated with CIN3+ were more common within a specific HPV31 lineage/sublineage, suggestive of lineage sorting due to genetic drift in non-recombining genomes (summarized in Table S8). One SNP mapping to the E7 oncogene (H23Y) was associated with CIN3+ (OR = 1.60, 95% CI = 1.17–2.19); this SNP was most common in samples with A2 or B2 genomes (Table S7). We performed this analysis for each lineage separately, but likely due to small numbers, no associations remained significant after correction for multiple tests.

We additionally evaluated whether the combined effect of rare (MAF < 0.01) synonymous and nonsynonymous variation by gene region was different between CIN3+ cases and controls. Considering all lineages together, L1 had more variation in controls (22.0%), compared to CIN3+ cases (16.8%) (Table 2). We stratified the analyses by HPV31 lineages and found that the A lineage controls had more variation across the genome (65.2%), and within E1 (19.6%) and L1 (23.3%) gene regions compared with the CIN3+ cases (genome = 58.3%, E1 = 8.7%, L1 = 12.6%) (Table 2). However, these differences did not remain significant after FDR correction. For B and C lineages, significant differences in rare variation were not observed between cases and controls (data not shown). For all lineages, the E7 oncogene region was least variable in both cases and controls. Interestingly, for the overall HPV31 population, L1 (dN/dS = 0.059, Z-value= −4.19, *p* < 0.001) and E1 (dN/dS = 0.119, Z-value = −3.48, *p* < 0.001) exhibited purifying selection (Table S9) consistent with their role as core proteins for the vegetative viral life cycle.

### 3.4. HPV31 Lineages Have Differing Methylation Levels

**Figure 3:**
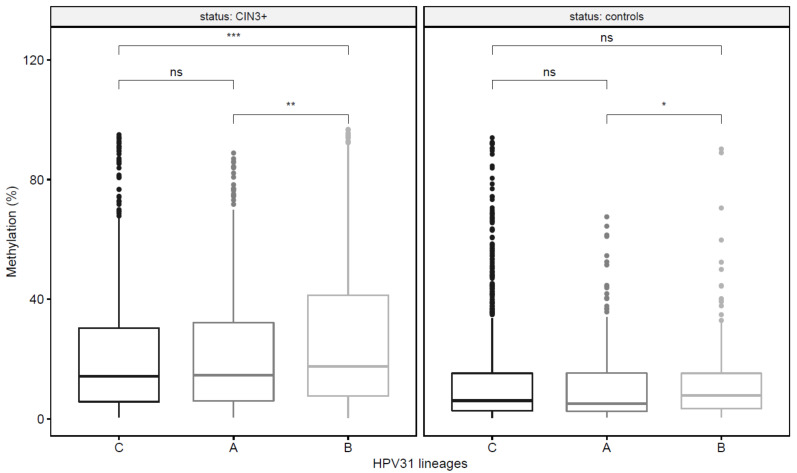
Viral methylation levels across all CpG sites tested by HPV31 lineages C, A and B, shown for CIN3+ cases and controls. The *y*-axis represents the percent methylation levels. *x*-axis represents each individual lineage. Footnote: *p*-values from Mann–Whitney (Wilcoxon Rank Sum) test. ns = non-significant; *** = *p* < 0.001; ** = *p* < 0.01; * = *p* < 0.05.

To evaluate whether HPV31 methylation levels differed by lineage and disease state, we tested 22 CpG sites within the E2, L1 and L2 gene regions [30], in a subset of 175 HPV31 PaP samples, including 89 CIN3+ cases and 86 controls. CpG methylation levels were compared between the HPV31 main lineages, A (*n* = 55), B (*n* = 41) and C (*n* = 79). The overall methylation across all 22 CpG sites varied between lineages and B had significantly higher methylation levels among controls (7.9 vs. A = 5.1, *p* = 0.03) and among CIN3+ cases (17.5 vs. A = 14.6, *p* < 0.01; vs. C = 14.2, *p* < 0.001) (Figure 3; Table S10). For the case-control comparisons, 20, 9 and 18 CpG sites in A, B and C lineages, respectively, had significantly higher methylation levels in CIN3+ cases compared to controls after correction for multiple tests (Table S11). The CpG site methylation level that best distinguished women with CIN3+ from controls, and had the strongest association with CIN3+, varied for each lineage: CpG site 3414 for women with HPV31 A (OR = 22.62, 95% CI = 5.2–99.2; AUC = 0.90), site 5530 for B (OR = 25.00, 3.4–184.5; AUC = 0.84), and site 5521 for C (OR = 7.20, 2.2–23.2, AUC = 0.80) (Table 3, Table S11).

## 4. Discussion

We report a large comprehensive evaluation of HPV31 genomic and epigenomic variation in relation to cervical carcinogenesis, using samples from two large studies within the U.S. and around the world. We show that lineages A and B and their sublineages had elevated association with cervical precancer and cancer compared to the C lineage. Sequencing over 2000 HPV31 genomes enabled us to identify specific individual SNPs, linked to HPV31 lineages, that were also associated with cervical precancer and cancer. The high methylation levels observed for the B lineage are consistent with the increased carcinogenicity of the A/B clade and a potential association with tissue dedifferentiation induced by viral gene products, where histologically differentiated cells regress to a less differentiated stage accompanied by epigenetic alterations of the virus that can in turn lead to uncontrolled epithelial cell replication and progression to cancer [54]. In addition, we identified different CpG sites to best distinguish CIN3+ cases from control infections for each of the main HPV31 lineages.

Viral genome sequence data allowed us to accurately determine HPV31 genetic variation down to the sublineage and nucleotide (i.e., SNP) level, and to investigate their association with precancer/cancer. We extend previously reported increased precancer risks associated with A and B lineages, compared to C [19,23]. Interestingly, our HPV31 phylogenetic tree indicated that the lineages most associated with CIN3+, A and B, share a common ancestor. Similarly, the carcinogenicity of HPV was first noted to be reflected by phylogenic relatedness for the 13 most common HR-HPV types from the alpha-5, -6, -7 and -9 species groups [6,7,55]. There was also variability in the CIN3+ associations at the sublineage level, with A1, A2 and B2 being up to 2.5-fold more likely to cause CIN3+, compared to the more common C3 sublineage. The individual SNP analyses confirmed the variability in the CIN3+ associations observed at the lineage/sublineage level, and SNPs linked to the C/C1/C2/C3/C4 lineage/sublineages were inversely associated with CIN3+, while SNPs linked to A/A1/A2/B2 lineage/sublineages were positively associated with CIN3+.

We also identified a new sublineage, C4, more common in women from Latin-America in the IARC study, but not the most common sublineage among Hispanic women nor significantly associated with CIN3+ among Hispanic women in the PaP cohort. The association of C4 and cervical carcinogenesis warrants further investigation in a larger sample size. The evolution and carcinogenesis of HPV lineages is not completely understood, since carcinogenesis neither facilitates viral replication nor transmission. Thus, based on significant differences in the geographic distribution of the HPV31 lineages, we surmise that genetic drift accounts for fixation of genetic changes. For HPV16 and HPV58, the time of divergence between some lineages corresponds to different out-of-Africa migration events from ~400,000 to ~100,000 years ago, as well as sexual transmission from archaic to modern humans [56,57], but this has not been evaluated for HPV31. Nevertheless, the genetic differences of HPV31 lineages associated with oncogenicity were likely acquired through long-term association with different host populations where features of enhanced viral fitness were under selection [58]. It has been suggested that the oncogenicity of HPVs are “collateral damage” after niche adaptation, particularly since the infectious virus is not made in precancer/cancer tissue. In fact, papillomaviruses that are associated with cervical neoplasia in macaques share a common ancestor with the oncogenic HPVs indicating a deep genotype-phenotype (e.g., niche adaptation) association with carcinogenicity [58,59]. Host immune alleles also likely play a role in selecting HPVs with carcinogenic potential possibly by extending viral persistence, given that specific HLA haplotypes have been associated with ICC [60].

We evaluated viral methylation at the lineage level and showed that CpG methylation was different by HPV31 lineages, similar to what has been observed at the HPV type level [18,26,27,30]. Here we focused on correlated CpG sites that were previously reported for HPV31 [30] and found higher methylation patterns across E2, L2 and L1 in CIN3+ cases compared to controls, using a large number of samples. Levels of methylation showed some variation by lineage and were increased for the B lineage compared to other lineages among both cases and controls. Viral methylation is associated with cervical precancer and cancer and is considered as a potential triage biomarker [18,27,30,61]. High methylation levels were correlated among CpG sites in L1 and L2, as well as E2, and these sites best distinguished precancers from controls, while methylation of CpG sites in the oncogenes E6/E7 and URR based on other studies did not differ for HPV31 cases and controls [18,30,32]. The functional impact of viral methylation on carcinogenesis remains elusive; it may be related to tissue dedifferentiation and lack of specific viral genes being transcribed in tissue no longer completing the full order of differentiation [62]. Given that viral methylation varies by HPV31 lineage, it will be important to evaluate methylation levels by other HR-HPV lineages to be certain that CpG sites with the strongest associations with CIN3+ across all lineages are included in future assays.

We observed that self-reported race/ethnicity modified the CIN3+ associations for HPV31 A lineage only among White women, compared to all other races/ethnicities in the PaP study. There was an indication for matching race/ethnicity and HPV31 geographic distribution between the C1 sublineage and African-American or women from Africa and, A2/C2 sublineages and Asian or women from Asia. All HPV31 lineages were found in cervical cancer samples across all world regions in the IARC study, likely a reflection of historical geographical dispersion. Perhaps due to small numbers, we did not see a clear pattern for matching of race/ethnicity with origins of the infecting virus and increased association with precancer/cancer as strongly as we previously observed for HPV16 [21,63] and HPV35 [64]. Self-reported race/ethnicity, obtained from the PaP Study, and worldwide distributions, obtained from IARC, are a proxy of human ancestry and geographic origin but do not precisely estimate the host’s genetic background, which is a limitation of our study. Future studies of host genetic ancestry using molecular genotype information may help to clarify these associations and elucidate interaction mechanisms of HPV carcinogenesis. We did not have duration of infection to investigate this point. There could also be other mechanisms at play, such as selection for an unmeasured phenotype of the virus that is associated with increased risk of epithelial transformation. An interaction between virus and host throughout thousands of years, along with human migration and reproductive events that resulted in introgression of immune related alleles from archaic hominins may have rendered specific HPV31 lineages the ability to persist and progress in some but not other human populations, as has been postulated for HPV16 [57,58].

At the SNP level, the T nucleotide (position 626) located in the E7 oncogene was most common in the A2/B2 sublineages and was associated with a 60% increased association with CIN3+. This is a nonsynonymous SNP resulting in a histidine (H) to tyrosine (Y) amino acid change at residue 23 of the E7 protein. This amino acid is located within the conserved region 2 (CR2) domain, specifically at the pRb binding site (Figure S2). The residue at position 23, part of the pRb-E7 core binding motif (21-XLXCXE-26) [65], is an important component for the E7-pRb bound conformation [66]. Interestingly, the Y amino acid (21-XLYCXE-26) is conserved in HPV16, but it is different in other papillomaviruses such as the *Alpha 10* HPV6 and HPV11 (21-XLHCXE-26), as well as the *Alpha 7* HPV18 (21-XLLCXE-26) [65]. A functional study has shown that a mutant version of the HPV6 E7 with the H23Y SNP has a higher affinity pRb binding site than the HPV6 wild type [67], leading to transactivation of the host’s cell cycle genes via the E2F transcription factor. We show that the H23Y (SNP at position 626) in E7, is more prevalent in the A2 and B2 sublineages and creates an identical 21-DLYCYE-26 pRb binding site to HPV16 E7, which suggests a shared carcinogenic component for both HPV31 A2/B2 and HPV16 given its higher pRb affinity. In vitro functional analyses with affinity measurements to investigate the effect of HPV31 A2/B2 21-DLYCYE-26 on pRb binding or degradation, as well as in other Rb family proteins such as p107 or p130 [68], would help to elucidate this for HPV31 A2/B2 lineages compared to HPV16 E7 [67]. Interestingly, A2 and B2 were among lineages most associated with CIN3+.

Rare genetic variation reflects more recent events in the evolutionary history of HPV and has also been linked to carcinogenesis [24]. The HPV31 E7 oncogene was the most hypovariable gene region for all HPV31 lineages, nevertheless we did not observe significant differences between cases and controls for HPV31 E7 variation in contrast to our observations for HPV16 [24]. Perhaps this relates to the lower carcinogenic potential of HPV31. We showed that controls had higher rare variation in the L1 gene, compared to CIN3+ cases, and for the A lineage, E1 and L1 had higher rare variation among controls compared to CIN3+ cases. Similar hypovariation in E1 and L1 in CIN3+ cases was also reported for HPV16 A1/A2 sublineages [24]. These findings might indicate that rare genetic variation of the oncogene, E7, is involved with different cancer risks associated with distinct HR-HPVs, but variation in genes such as E1 and L1 may be related to the ability of the virus to persist, and eventually lead to cancer. In fact, viral persistence has been associated with subsequent increased risk of progression to cancer [69,70]. E1 is one of the most conserved genes among HPVs with unique enzymatic activity involved in viral replication in the first phase of infection [71]. Therefore, it is possible that genetic variation within E1 may alter interactions with the host replication machinery in the nucleus, resulting in different levels of viral replication and/or persistence. L1, the protein which the vaccine is based upon [72], is the major capsid protein forming the exterior surface of the virus and the first point of contact with host cells, important for the infectious viral life cycle phase [73], but its function in persistence needs to be clarified. Here, we did not have longitudinal data to assess genomic variation and persistence throughout infection, but we plan to assess this in our follow-up studies. Other factors such as HPV31 within-host viral diversity and viral somatic variants may influence carcinogenesis, as shown for HPV16 [74], warranting further investigation.

## 5. Conclusions

We have amassed the largest study of HPV31 genomic variation and methylation to date. Nucleotide variation and increased methylation may serve as markers for identification of CIN3+ lesions in women infected with HPV31. Finer levels of viral genetic variation, including sublineages and SNPs, as well as methylation patterns, influence the relationship between HPV31 and cervical carcinogenesis. The distribution of HPV31 lineages/sublineages vary by race/ethnicity and geographic origins of populations. This supports the notion that viral–host interaction over the last few hundred thousand years has resulted in some type of adaption of the virus to the host. This is most clearly reflected in the increased associations with carcinogenicity for a common subtype amongst certain disparate population groups.

## Figures and Tables

**Table 1:** HPV31 lineage associations with CIN3+, and effect modification of race/ethnicity, in the PaP cohort.

Lineages	Controls		CIN3+		OR	95% CI		*p*
	N	%	N	%				
*Lineage*								
C	521	50.4%	89	35.6%	ref			
A	326	31.5%	103	41.2%	**1.85**	**1.35**	**2.54**	
B	187	18.1%	58	23.2%	**1.82**	**1.25**	**2.63**	
Total	1034	100.0%	250	100.0%				
*Sublineage*								
C3	322	31.1%	55	22.0%	ref			
A1	267	25.8%	78	31.2%	**1.71**	**1.17**	**2.5**	
A2	59	5.7%	25	10.0%	**2.48**	**1.43**	**4.29**	
B1	35	3.4%	9	3.6%	1.51	0.69	3.31	
B2	152	14.7%	49	19.6%	**1.89**	**1.23**	**2.9**	
C1	58	5.6%	6	2.4%	0.61	0.25	1.47	
C2	37	3.6%	4	1.6%	0.63	0.22	1.85	
C4	104	10.1%	24	9.6%	1.35	0.8	2.29	
Total	1034	100.0%	250	100.0%				
*Race/Ethnicity versus all others*								
*A Lineage*								
White	143	50.4%	64	63.4%	**1.71**	**1.07**	**2.72**	
Hispanic	81	28.5%	19	18.8%	0.58	0.33	1.02	
African-American	18	6.3%	3	3.0%	0.45	0.13	1.57	
Asian	42	14.8%	15	14.9%	1.00	0.53	1.90	**0.017**
Total	284	100.0%	101	100.0%				
*B Lineage*								
White	90	54.9%	29	53.7%	0.95	0.51	1.77	
Hispanic	42	25.6%	15	27.8%	1.12	0.56	2.23	
African-American	11	6.7%	3	5.6%	0.82	0.22	3.05	
Asian	21	12.8%	7	13.0%	1.01	0.41	2.54	0.976
Total	164	100.0%	54	100.0%				
*C Lineage*								
White	237	49.0%	48	57.1%	1.39	0.87	2.22	
Hispanic	132	27.3%	16	19.0%	0.63	0.35	1.12	
African-American	51	10.5%	6	7.1%	0.65	0.27	1.57	
Asian	64	13.2%	14	16.7%	1.31	0.7	2.47	0.113
Total	484	100.0%	84	100.0%				

Footnote: Controls = Cervical intraepithelial neoplasia (CIN) grade 1 or lower; CIN3+ = CIN grade 3 and cancer; OR = Odds ratio and 95% confidence intervals (CI) from logistic regression; For race/ethnicity, the reference group is all other races combined except the tested group. *p =* Wald-test for heterogeneity. Significant *p*-values are bolded.

**Table 2:** Rare variant burden analysis for all HPV31 lineages, and within the A lineage, in the PaP Cohort.

Viral Gene/Region	No. Individuals with Variants (%)				*p*	*p*-FD*R*
	All HPV31 lineages (*n* = 1284)					
	Controls (*n* = 1034)		CIN3+ cases (*n* = 250)			
E1	223	21.6%	45	18.0%	0.214	0.535
E2	148	14.3%	34	13.6%	0.772	0.875
E4	65	6.3%	8	3.2%	0.064	0.320
E5	58	5.6%	13	5.2%	0.799	0.875
E6	61	5.9%	12	4.8%	0.501	0.835
E7	21	2.0%	3	1.2%	0.389	0.778
L1	236	22.8%	42	16.8%	**0.039**	0.320
L2	375	36.3%	92	36.8%	0.875	0.875
URR	181	17.5%	41	16.4%	0.679	0.875
WG	724	70.0%	164	65.6%	0.175	0.535
	**HPV31 A lineages (*n* = 429)**					
	**Controls (*n* = 326)**		**CIN3+ cases (*n* = 103)**			
E1	61	18.7%	9	8.7%	**0.020**	0.163
E2	35	10.7%	6	5.8%	0.146	0.292
E4	19	5.8%	1	1.0%	0.074	0.185
E5	13	4.0%	2	1.9%	0.335	0.497
E6	12	3.7%	6	5.8%	0.348	0.497
E7	5	1.5%	1	1.0%	0.674	0.674
L1	74	22.7%	14	13.6%	**0.049**	0.163
L2	114	35.0%	39	37.9%	0.593	0.672
URR	53	16.3%	19	18.4%	0.605	0.672
WG	225	69.0%	60	58.3%	**0.045**	0.163

Footnote: Controls = Cervical intraepithelial neoplasia (CIN) grade 1 or lower; CIN3+ = CIN grade 3 and cancer; L1 = Late gene 1; L2 = Late gene 2; E1 = early gene 1; E2 = early gene 2; E4 = early gene 4; E5 = early gene 5; E6 = early gene 6; E7 = early gene 7; URR = untranslated regulatory region; WG = whole HPV31 genome. *p* = Fisher’s exact test. Significant *p*-values are bolded.

**Table 3:** Top CpG sites with high methylation associated with CIN3+ for each HPV31 lineage in the PaP Cohort.

Gene	Lineage	CpG Site ^†^	Controls (*n* = 85)		CIN3+ (*n* = 89)		Difference	p *	*p*-FDR	AUC	95% CI		OR	95% CI		*p ^#^*	*p*-FDR
			*n*	Median	*n*	Median											
E2	A	3414	22	1.89	32	6.19	4.30 **	6.6 × 10^−7^	4.4 × 10^−5^	0.90	0.82	0.98	22.62	5.16	99.19	3.5 × 10^−5^	0.001
	B	3414	13	5.47	28	7.77	2.30	0.560	0.560	0.56	0.37	0.74	1.81	0.47	6.97	0.390	0.410
	C	3414	50	2.67	29	5.29	2.62	0.035	0.049	0.64	0.52	0.77	3.38	1.29	8.81	0.013	0.020
L2	A	5530	22	3.97	32	11.88	7.92	0.005	0.011	0.73	0.58	0.88	9.52	2.71	33.51	4.5 × 10^−4^	0.003
	B	5530	9	4.33	27	19.41	15.08 **	0.002	0.007	0.84	0.66	1.00	25.00	3.39	184.50	0.002	0.004
	C	5530	45	4.32	23	11.56	7.24	0.004	0.009	0.72	0.59	0.85	5.14	1.69	15.63	0.004	0.007
L2	A	5521	22	2.66	32	5.40	2.74	0.004	0.009	0.73	0.58	0.88	8.00	2.33	27.46	9.5 × 10^−4^	0.004
	B	5521	9	3.65	27	6.62	2.97	0.096	0.110	0.69	0.46	0.91	5.50	1.07	28.20	0.041	0.056
	C	5521	45	3.10	23	6.26	3.16 **	6.0 × 10^−5^	0.002	0.80	0.69	0.91	7.20	2.24	23.17	9.3 × 10^−4^	0.004

Footnote: Controls = Cervical intraepithelial neoplasia (CIN) grade 1 or lower; CIN3+ = CIN grade 3 and cancer; † = HPV31 genome position; ** = Sites that best distinguished cases from controls for each lineage; *p* * = Mann–Whitney (Wilcoxon Rank Sum) *p*-value; *p* ^#^ = Univariate regression *p*-value; *p*-FDR = Adjusted false discovery rate *p*-value; AUC = Area under the curve; OR = Odds ratio and 95% confidence intervals (CI) for the association between high methylation, dichotomized at the 2nd tertile based on controls for each site, and CIN3+, with controls as the referent.

## Data Availability

The HPV31 sequenced genomes from samples assessed in both the genomic and epigenomic analyses have been uploaded to GenBank and are available under accession numbers MT750511-MT752599 and MT752601-MT752604. Other variables that support the findings of this study are available from the corresponding author upon request.

## Supplementary Materials

The following materials are available online at https://www.mdpi.com/article/10.3390/v13101948/s1, Figure S1. Analytical datasets of all analyses related to HPV31 infection from the PaP Cohort and IARC Biobank. Figure S2. Amino-acid alignment with schematic representation of E7 structure and domains, with reference protein sequences from HPV16, 31 and 35 obtained from the Papillomavirus Episteme database (PaVE). Table S1. Genome references used to build phylogenetic tree and perform lineage assignment. Table S2. Methylation assay primer sequences. Table S3. Summary of sequencing coverage and quality statistics of the HPV next generation sequencing data. Table S4. Characteristics of HPV31-positive samples from the PaP cohort and IARC studies. Footnote: CIN2 = Cervical intraepithelial neoplasia (CIN) grade 2; CIN3 = CIN grade 3; AIS = adenocarcinoma *in situ*; HSIL = High-grade squamous intraepithelial lesion. Table S5. IARC HPV31-positive sample collection by region/country, status and sublineage. Footnote: 1. “Control”, “HSIL/CIN2/3” and “unknown histology” are included. Table S6. HPV31 lineage/sublineage distribution among samples from the PaP Cohort and IARC. Footnote: ≤ CIN1 = Cervical intraepithelial neoplasia (CIN) grade 1 or lower; CIN3+ = CIN grade 3 and cancer; *p* *= Fisher’s exact test using Monte Carlo simulation; *p ^#^* = Chi-square test. Significant *p*-values are bolded. ^⌘^For the PaP Cohort, 138 women were self-reported as multiple race/ethnicities or no race/ethnicity was available. Table S7. HPV31 lineage associations with CIN2+, and effect modification of race/ethnicity, in the PaP cohort. Footnote: ≤ CIN1 = Cervical intraepithelial neoplasia (CIN) grade 1 or lower; CIN2+ = CIN grade 2, grade 3 and cancer; OR = Odds ratio and 95% confidence intervals (CI) from logistic regression; For race/ethnicity, the reference group is all other races combined but the tested group. A total of 138 women did not report race/ethnicity or reported as from multiple races. *p*=Wald-test for heterogeneity. Significant *p*-values are bolded. Table S8. Associations between CIN3+ and individual SNPs in the PaP cohort. Footnote: NC = nucleotide; AA = amino-acid; TriNC = trinucleotide within a codon in which SNPs are located; *variants potentially induced by APOBEC with motifs 5′[T] [C→T or C→G] [A or T]3′; ^#^Frequency of each SNP within a particular lineage; OR = Odds ratio and 95% confidence intervals (CI) from logistic regression; *p*-FDR = False discovery rate (FDR) correction was performed for the number of common mutations with MAF > 1%, *n* = 199; Significant *p*-values are bolded. Table S9. Non-synonymous over synonymous mutation rate by HPV gene region in PaP cohort. Footnote. Analyses performed with SNPgenie (Nelson and Hughes, 2015 (https://github.com/chasewnelson/SNPGenie)). Significant *p*-values are bolded. Table S10. Comparison of methylation levels across HPV31 lineages. *p* * = Mann–Whitney (Wilcoxon Rank Sum) *p*-value. Significant *p*-values are bolded. Table S11. Methylation levels at each CpG site and associations with CIN3+ for each HPV31 lineage. Footnote: † = HPV31 genome position; *p* * = Mann–Whitney (Wilcoxon Rank Sum) *p*-value; *p*^#^ = Univariate Regression; AUC = Area under the curve; OR = Odds ratio and 95% confidence intervals (CI) for the association between high methylation, dichotomized at the 2nd tertile based on controls for each site, and CIN3+, with controls as the referent.

## Abbreviations

AIS	adenocarcinoma in situ
AUC	areas under the curve
CI	confidence intervals
CIN	cervical intraepithelial neoplasia
CIN1	cervical intraepithelial neoplasia grade 1
CIN	cervical intraepithelial neoplasia grade 2
CIN2+	cervical intraepithelial neoplasia grade 2, grade 3 and cancer
CIN3	cervical intraepithelial neoplasia grade 3
CIN3+	cervical intraepithelial neoplasia grade 3 and cancer
DNA	deoxyribonucleic acid
E1	early gene 1
E7	early gene 7
FDR	false discovery rate
FFPE	formalin fixed paraffin-embedded
HC2	Hybrid Capture 2
HPV	Human papillomavirus
HPV31	Human papillomavirus type 31
HR	high-risk
IARC	International Agency for Research on Cancer
ICC	invasive cervical cancer
KPNC	Kaiser Permanente Northern California
L1	late gene 1
MAF	minor allele frequency
MRCA	most recent common ancestor
NCI	National Cancer Institute
OR	odds ratio
PaP	Persistence and Progression
PCR	polymerase chain reaction
ROC	Receiver operating characteristic
SNPs	single nucleotide polymorphisms
STM	specimen transport medium
vs.	versus
