# Supplementary material for: Phylogenomic Analysis of Human Papillomavirus Type 31 and Cervical Carcinogenesis: A Study of 2093 Viral Genomes
Source: Viruses. 2021 Sep 28;13(10):1948. doi: 10.3390/v13101948 (PMC8540939; doi:10.3390/v13101948)
Supplement: Supplementary file 1 [file viruses-13-01948-s001.zip › Supplemental figure S1.pdf]

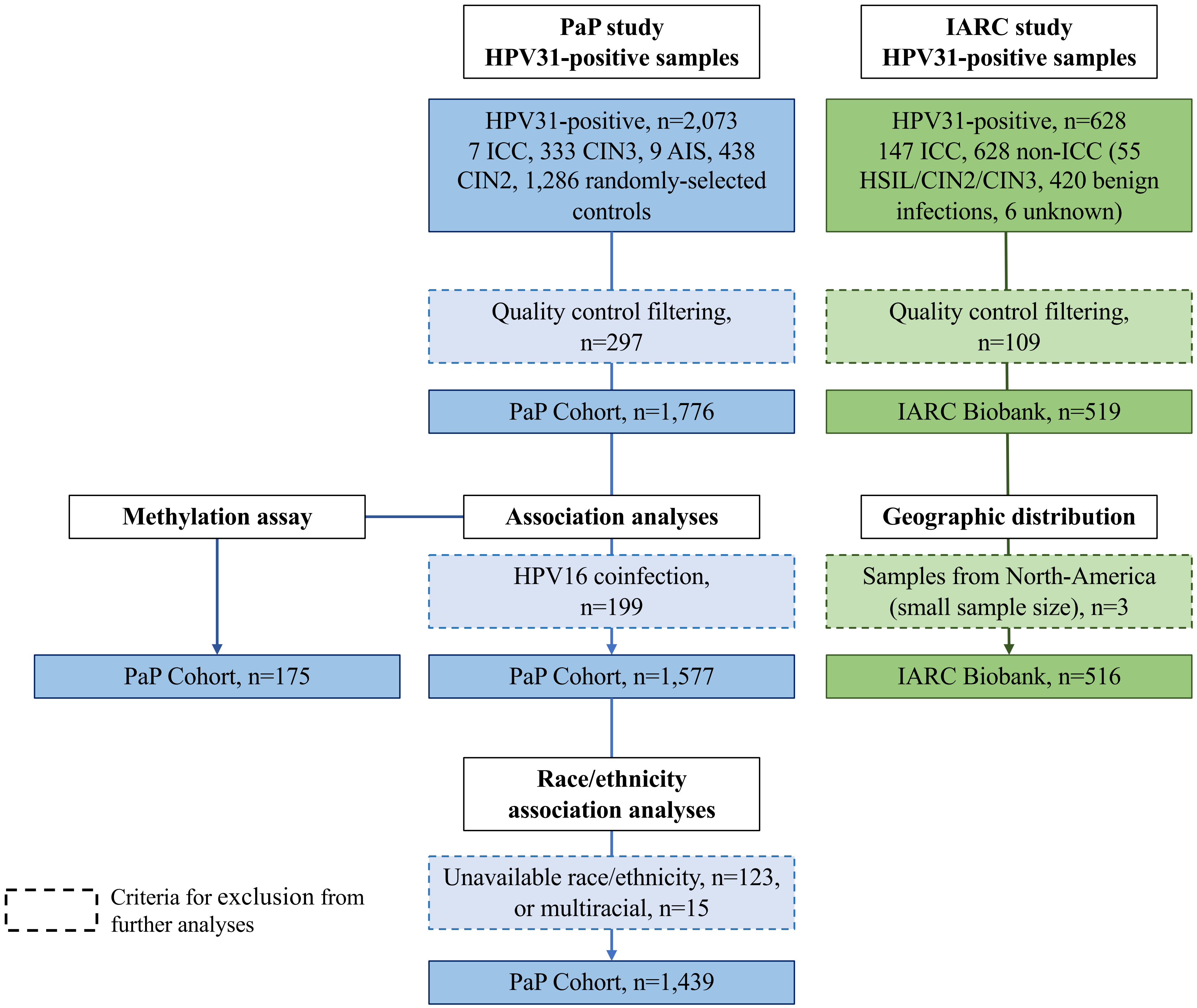

Figure S1. Analytical datasets of all analyses related to HPV31 infection from the PaP Cohort and IARC Biobank
