# Supplementary material for: Phylogenomic Analysis of Human Papillomavirus Type 31 and Cervical Carcinogenesis: A Study of 2093 Viral Genomes
Source: Viruses. 2021 Sep 28;13(10):1948. doi: 10.3390/v13101948 (PMC8540939; doi:10.3390/v13101948)
Supplement: Supplementary file 1 [file viruses-13-01948-s001.zip › Supplemental Figure S2.pdf]

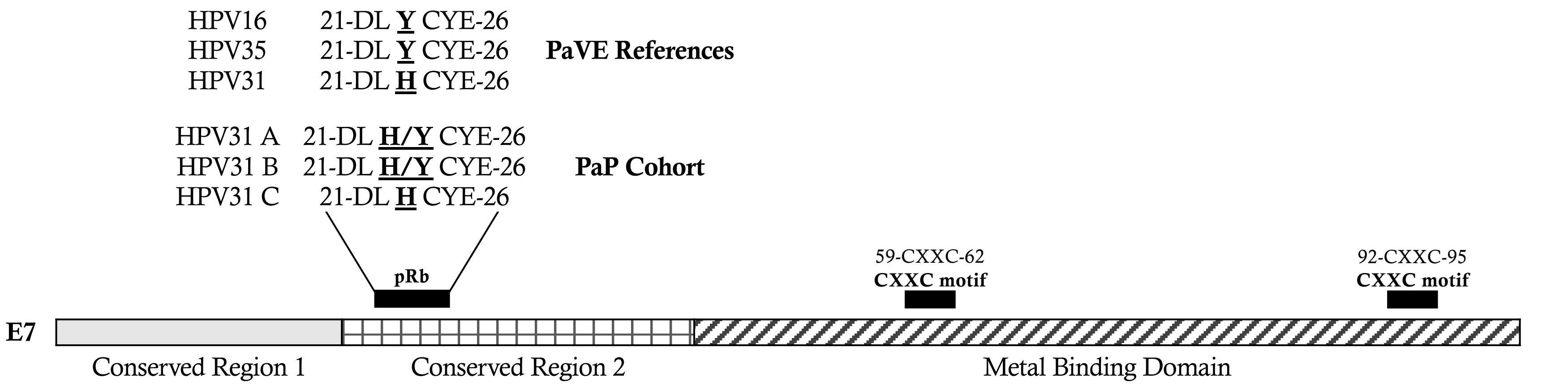

Figure S2. Amino-acid alignment with schematic representation of E7 structure and domains, with reference protein sequences from HPV16, 31 and 35 obtained from the Papillomavirus Episteme database (PaVE).
